# Evaluating the Accuracy of the Azure Kinect and Kinect v2

**DOI:** 10.3390/s22072469

**Published:** 2022-03-23

**Authors:** Gregorij Kurillo, Evan Hemingway, Mu-Lin Cheng, Louis Cheng

**Affiliations:** 1 Bioniks, Alameda, CA 94501, USA; mulinc@uci.edu (M.-L.C.); lcheng@bioniks.net (L.C.); 2 Applied BioMechanics, Alameda, CA 94501, USA; ehemingway@appliedbiomech.com

**Keywords:** 3D sensing, Azure Kinect, depth accuracy, Kinect v2

## Abstract

The Azure Kinect represents the latest generation of Microsoft Kinect depth cameras. Of interest in this article is the depth and spatial accuracy of the Azure Kinect and how it compares to its predecessor, the Kinect v2. In one experiment, the two sensors are used to capture a planar whiteboard at 15 locations in a grid pattern with laser scanner data serving as ground truth. A set of histograms reveals the temporal-based random depth error inherent in each Kinect. Additionally, a two-dimensional cone of accuracy illustrates the systematic spatial error. At distances greater than 2.5 m, we find the Azure Kinect to have improved accuracy in both spatial and temporal domains as compared to the Kinect v2, while for distances less than 2.5 m, the spatial and temporal accuracies were found to be comparable. In another experiment, we compare the distribution of random depth error between each Kinect sensor by capturing a flat wall across the field of view in horizontal and vertical directions. We find the Azure Kinect to have improved temporal accuracy over the Kinect v2 in the range of 2.5 to 3.5 m for measurements close to the optical axis. The results indicate that the Azure Kinect is a suitable substitute for Kinect v2 in 3D scanning applications.

## 1. Introduction

Depth sensing is important in various applications, such as augmented and virtual reality (AR/VR) [1], human–computer interaction [2], robotic manipulation [3], and navigation [4]. Whereas the early applications of depth sensing relied on passive imaging from stereo cameras and structure from motion, advances in sensor technologies and computing have enabled the development of low-power and compact active sensing solutions (see the survey of commercial 3D cameras in [5]).

The release of the Microsoft Kinect Sensor for Xbox 360 (a.k.a. Kinect v1) in 2010 brought depth sensing to the mainstream. The Kinect v1 incorporated a depth sensor, a color camera, and a four-microphone array that provided full-body 3D motion capture, facial recognition, and voice recognition capabilities [6]. The depth sensing of Kinect v1 was based on the structured light principle, acquired from the projected infrared (IR) dot pattern and IR camera. Although the Kinect was originally purposed for gaming and entertainment, the research community embraced it as a new low-cost solution for applications ranging from 3D surface reconstruction to healthcare.

In 2012 and 2013, Microsoft released updated versions of their Kinect sensor as Kinect for Windows and Kinect for Xbox One (a.k.a. Kinect v2), respectively. The depth sensing of the Kinect v2 is based on the time of flight (ToF) principle, where the distance of the observed surface point is acquired by computing the round-trip time of a pulsating IR laser light [7]. Using ToF technology for depth sensing in the Kinect v2 provided several advantages over the previous generation, including higher output resolution of the depth data [8], a wider field of view for both color and depth cameras, lower noise level, less sensitivity to lighting, and more constant depth measurement precision across the operating range [9]. Due to low adoption of the Kinect v2 by game developers and consumers, the production of the sensor was discontinued in 2017.

In 2020, Microsoft released their newest version of the sensor as the Azure Kinect DK (Development Kit), which is marketed to enterprise users in areas such as retail, manufacturing, robotics, and healthcare. The development kit includes software support for data acquisition, body tracking, and integration with cloud-based artificial intelligence (AI) services offered by the Microsoft Azure cloud platform. Currently, the Azure Kinect hardware includes: a 12-megapixel color camera, a 1-megapixel ToF depth camera, an inertial measurement unit (IMU), and a seven-microphone circular array [10]. The updated depth sensor provides greater resolution, a wider field of view, a global shutter, pixel binning, and reduced power consumption [11]. Furthermore, the software support includes Windows and Linux platforms making the device attractive for cross-platform development.

Since many of the developers that were using Kinect v2 are transitioning to Azure Kinect, this paper focuses on comparing the overall accuracy between the two latest generations of the Kinect sensor. We first review prior work on the accuracy evaluation of the Kinect v2 and Azure Kinect. Next, we describe the methodology for our accuracy assessment using two different methods. We then present the results for the two Kinect systems using FARO laser scanner data as ground truth and a side-by-side comparison of the temporal error analysis across the field of view of two sensors. Finally, we conclude with a discussion of our accuracy assessment.

## 2. Related Work

The accuracy of the Kinect v2 has been a topic of several research papers since its initial release in 2012. Gonzalez-Jorge et al. [12] compared the accuracy of the Kinect v1 and Kinect v2 point cloud data by capturing a manufactured object consisting of five spheres and seven cubes of known dimensions. The evaluation was performed at a range of 1.0 to 4.0 m with 1 m intervals and at three different angles. Precision was then evaluated using the standard deviation of the least squares algorithm applied to the fitting of the spheres and the top face of the largest cube.

Lachat et al. [13] reported on the accuracy of the Kinect v2 for close-range measurements by evaluating the standard deviation of collected depth data when the camera was positioned parallel to a white planar wall. The authors noted that the camera has a pre-heating time of about 30 min, after which the noise level stabilizes. Overall standard deviation of the range measurements when observing a planar surface was reported to be between 2 and 5 mm at 1.2 m. The study also compared the Kinect v2 acquisition with a FARO laser scanner for small object scanning.

Fankhauser et al. [4] examined the systematic errors of the Kinect v2 camera for mobile navigation. In their work, they evaluated depth distortion and amplitude-related errors when the camera observed a flat target at different angles and distances. They determined that depth distortion follows an oscillatory pattern over the range of various distances. The depth distortion caused by the irregularities in the modulation process of the illuminator was found to result in errors from ±6 mm around the center and up to 30 mm toward the edges of the image at the typical usage distance of 2.0 m.

A study by Pagliari and Pinto [9] proposed a calibration method of the Kinect v2 depth camera to reduce the error of the depth camera due to lens distortion. The depth accuracy was assessed by analyzing the standard deviation of the range measurements across 100 images of an indoor static scene and a flat surface placed at various distances from the camera. The authors found that the standard deviation was mostly under 2 mm at 1.5 m but increased at the corners of the field of view.

Yang et al. [7] used a laser range finder to evaluate the absolute accuracy of the depth capture. A planar surface was placed at various key positions in space, while Kinect v2 and laser data were captured. The laser provided true depth value measurements. The researchers obtained a three-dimensional cone model that described the spatial distribution of the average depth accuracy. An average depth accuracy error of under 2 mm was observed in the central region of the cone for the ranges from 0.5 to 3.0 m, 2 to 4 mm for ranges from 3.0 to 3.5 m, and over 4 mm for distances beyond 3.5 m.

Accuracy evaluations of the Azure Kinect have been limited to date. Tölgyessy et al. [14] compared Azure Kinect with Kinect v1 and v2 in terms of depth noise for objects of different reflectivity. The authors reported that, overall, the observed depth noise in the Azure Kinect was twice as small as compared to the Kinect v2 for distances under 3 m in the near field of view (NFOV) mode and comparable to the Kinect v2 in its wide field of view (WFOV) mode. The report further analyzed several other aspects of the Azure Kinect, including warm-up time, precision, accuracy, reflectivity, and the multipath and flying pixel phenomena. The depth accuracy of the camera was obtained by mounting a white reflective plate to a robotic manipulator and positioned at various distances from the camera. Although the robotic manipulator can provide high positioning accuracy, no absolute baseline measurement of the plate was obtained, other than the starting point obtained through the camera itself. Furthermore, the depth accuracy was only evaluated along the optical axis of the sensor.

For completeness, we list two application-specific accuracy evaluations that have employed the Azure Kinect, primarily for dimensional measurements without providing a general accuracy assessment. McGlade et al. [15] evaluated the Azure Kinect to measure tree stem diameter from acquired point clouds. Neupane et al. [16] used the Azure Kinect (among other commercial depth sensors) to assess its accuracy for distance and size measurements of fruit in orchards. There have been several other publications involving the Azure Kinect related to human posture tracking, which is beyond the scope of this work.

This paper aims to fill the gap in accuracy assessments of the new Azure Kinect by providing a benchmark study with a laser scanner and Kinect v2 camera system for comparison. Although the recent report by Tölgyessy et al. [14] provides an extensive overview of various aspects of the sensor, their work lacks information on the absolute accuracy across the field of view of the camera. Our experimental methodology follows the study by Yang et al. [7] that was performed using the Kinect v2. As opposed to Yang’s study, which evaluated distance at a single point, we use a laser scanner to obtain the ground truth for the absolute location of the target geometry.

## 3. Materials and Methods

### 3.1. Hardware

The Microsoft Azure Kinect and Kinect v2 were evaluated for depth and spatial accuracy against ground truth data provided by a FARO laser scanner. More details on the sensors and their configurations are provided below. A summary of their characteristics may be found in Table 1.

#### 3.1.1. Kinect v2

The Kinect v2 hardware used in this study was the Kinect for Xbox version with Kinect for Windows USB3 adapter (Microsoft Inc., Redmond, WA, USA). The Kinect v2 features an HD color camera (1920 × 1080 px) and a ToF depth sensor (512 × 424 px) with high dynamic range that delivers data at 30 frames per second (FPS). The depth sensor operates with multi-frequency photo-demodulation (up to 130 MHz) to achieve the high dynamic range and suppress the effects of visible light on the output [8]. The Kinect v2 depth sensor specifications include a field of view (FOV) of 70° × 60° and an operating range from 0.5 to 4.5 m. This results in a ground sample distance (GSD) between 1.4 mm at 0.5 m range and 12 mm at 4.5 m range [13].

#### 3.1.2. Azure Kinect

The Azure Kinect DK (Microsoft Inc., Redmond, WA, USA) includes a 12-megapixel color camera (4096 × 3072 px) and a 1-megapixel ToF depth sensor (1024 × 1024 px). Its ToF sensor features a global shutter with analog binning that provides pixel-synchronized capture with reduced noise in lower-resolution image modes. The sensor operates with a modulation frequency from 200 to 320 MHz, achieving similar dynamic range to the Kinect v2 [11]. Additionally, the device supports several operating modes that differ in resolution, range, and maximal frame rate.

The maximal FOV of the depth camera is 120° × 120° in WFOV mode and 75° × 65° in NFOV mode. In WFOV mode, the maximum output resolution is 1024 × 1024 px unbinned and 512 × 512 px binned. The operating range in WFOV mode is 0.25 to 2.21 m (0.25 to 2.88 m binned), while the range in the NFOV mode is 0.50 to 3.86 m (0.50 to 5.46 m binned). In NFOV mode, the maximum output resolution is 640 × 576 px unbinned and 320 × 288 px binned. According to the hardware specifications [10], the random depth error is ≤17 mm, while the systematic depth error is <11 mm + 0.1% of distance without multi-path interference.

Since our goal was to compare the performance of the Azure Kinect vs. Kinect v2, we evaluated the measurement accuracy in the mode that is comparable to the Kinect v2 capture specifications. The data reported in this paper were therefore obtained in the NFOV mode without binning, with the output color resolution of 1920 × 1080 px. These options are the most likely choice for existing applications that intend to upgrade to the next generation sensor with the data acquisition rate of 30 FPS.

#### 3.1.3. FARO Laser Scanner

The benchmark data for the accuracy evaluation were obtained using the X330 HDR FARO 3D Laser Scanner (FARO Technologies, Inc., Lake Mary, FL, USA). To capture point data, the laser scanner sends an infrared laser beam into the center of its rotating mirror. The mirror deflects the laser beam around the environment being scanned, while the scattered light is reflected back to the scanner. By measuring the phase shift of the light waves and the current angles of the mirror with respect to the sensor, the 3D coordinates of each point are computed with high accuracy. The single point measurements are repeated up to 976,000 times per second [17]. The range of FARO X330 HDR laser scanner is 0.6 to 330 m, with widest fields of view of 0 to 360° and −60 to 90° in the horizontal and vertical directions, respectively. For this data collection, the capture in the horizontal direction was limited to a range of 0 to 180° and in the vertical direction of −62.5 to 90°. The scan profile used was set to “Indoor … 10 m” (0.6 to 10 m range) with the following parameters: quality 3x, resolution 5.6 million points, scan size 2560 × 2170 points. Under these settings, the scan time was approximately 3 min 33 s. All our measurements were performed indoors at the range below 10 m.

Per product specifications of the maximum ranging error for this model is within ±2 mm, defined as a systematic measurement error at 10 and 25 m distances. The ranging noise, defined as the standard deviation of values about the best-fit plane for a measurement speed of 122,000 points/second, is reported to be 0.3 mm at this range.

The scanner unit used in this study was calibrated by FARO less than a year prior to data collection. The uncertainty reported in the calibration certificate was 0.496 mm (one sigma) at the distance of 9.8096 m, with a deviation of −0.2 mm at a distance of 9.8098 m.

### 3.2. Setup and Data Collection: Flat Board

**Figure 1:**
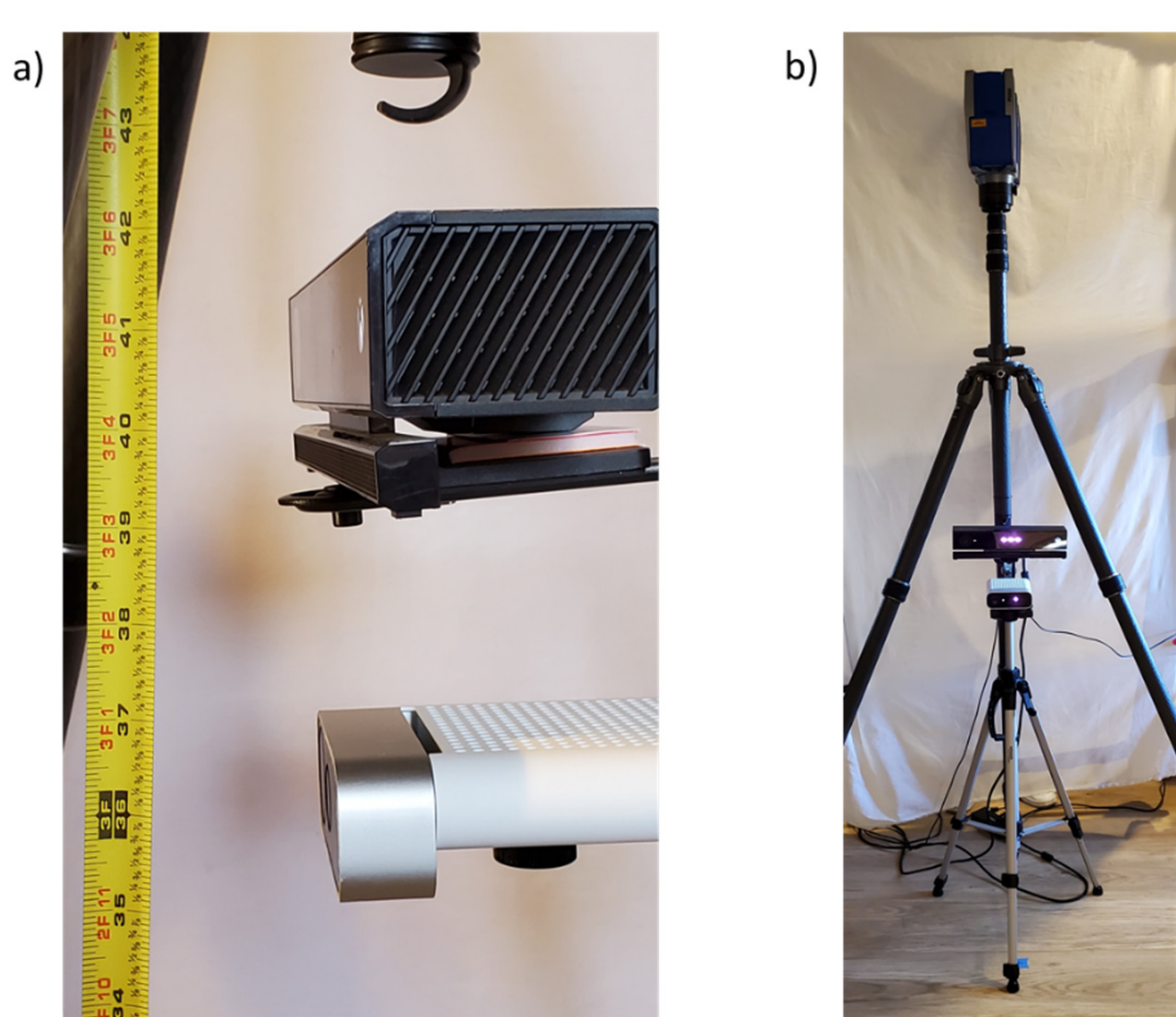
(**a**) The Kinect v2 (above) and Kinect Azure (below) are shown with a tape measure positioned vertically from the ground up for reference, and (**b**) both Kinect sensors are mounted on a tripod with a FARO 3D laser scanner positioned directly above.

A 6.1 × 4.9 m (20 × 16 ft) indoor space was used as the environment for testing. The room was illuminated with LED ceiling fixtures, and the curtains were drawn to block any direct sunlight in order to minimize its effect on sensor precision. The Kinect v2 and Azure Kinect were mounted vertically adjacent to one another on the same tripod (Figure 1) and placed at one end of the room. The midpoint between the two Kinect devices was set at an elevation of about 98 cm (38.5 in) from the ground floor. The cameras were aligned vertically such that their frontal surfaces were approximately in the same plane. The cameras were otherwise centered on the tripod, and their tilt and pitch were zeroed manually using a level. The FARO laser scanner was placed on a tripod directly above the Kinect devices, placing the height of the scanner axis at about 189.2 cm (74.5 in) from the ground plane.

**Figure 2:**
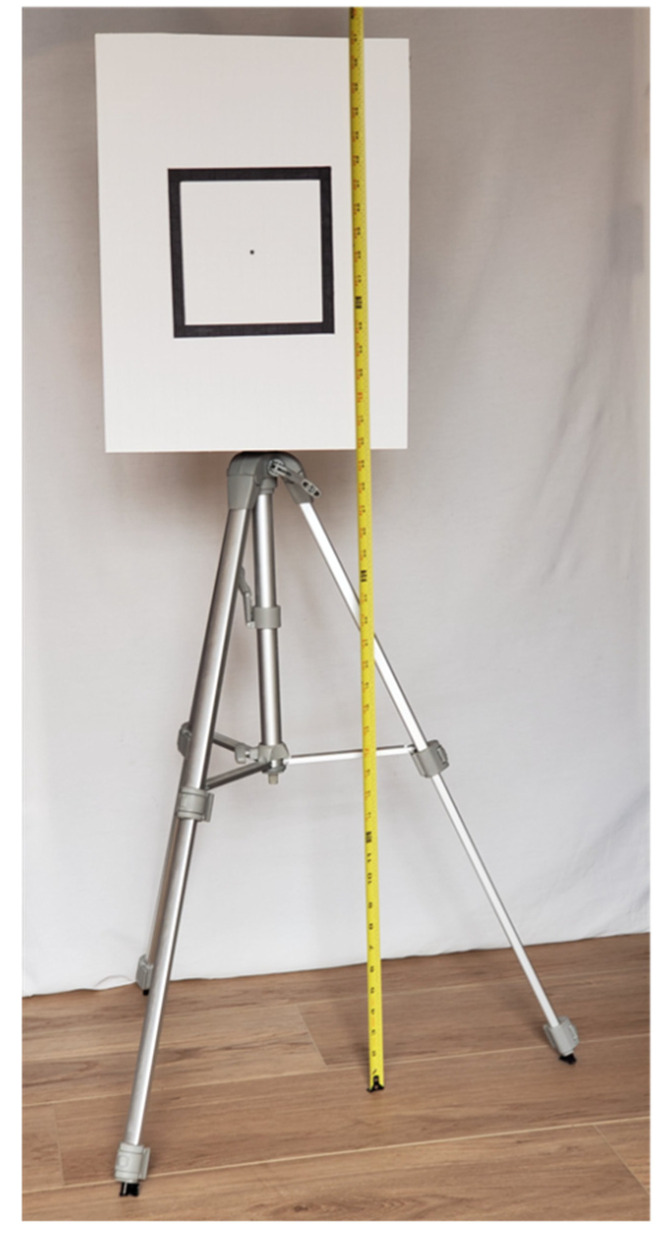
A planar board marked with a rectangle and a point is used as the test object for scanning.

A planar surface was evaluated in this study (Figure 2). The plane was represented by a rectangular surface of about 33.7 × 43.8 cm (13.25 × 17.25 in) in size. A flat board about 6.4 mm (0.25 in) thick and made of engineered wood was coated with a white non-reflective paper. A rectangle of about 17.8 × 17.8 cm (7 × 7 in) was marked at the center of the board with a black marker. The board was mounted on a tripod with the midpoint located at about 98 cm (38.5 in) from the ground floor and aligned vertically with a level.

**Figure 3:**
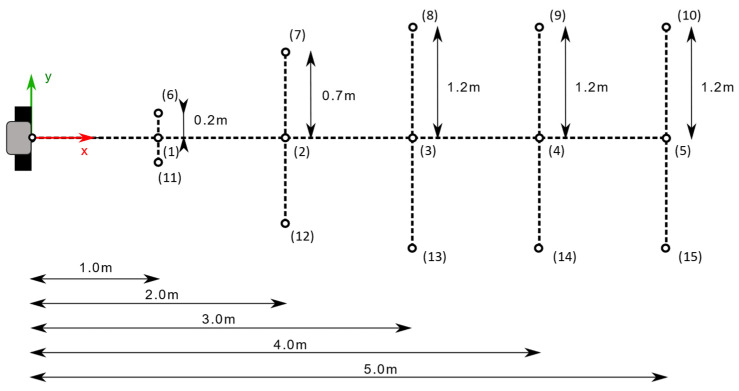
The grid arrangement of the target object locations for testing. Numbers in parentheses denote the location identifiers. Kinect cameras and the laser scanner are located at the origin of the coordinate system. The Kinect cameras are in the same orientation for every target location, such that the optical axis of either camera is always aligned with the *x*-axis.

In a manner similar to that followed in the study by Yang et al. [7], the object was placed at several locations in the field of view to assess the accuracy of the depth acquisition by the two sensors. The floor in front of the cameras was partitioned into a grid as shown in Figure 3. The locations were measured from the point on the floor directly underneath the cameras and marked with tape. The planar object was kept at the same height for all scans and was roughly oriented such that the board normal pointed toward the Kinect cameras.

The data collection from each Kinect device was performed simultaneously using Brekel PointCloud v3 software (Brekel, Amsterdam, The Netherlands), which provides the capture of raw Kinect data (i.e., color and depth images) and the reconstruction of 3D point cloud. Both devices were connected to the same laptop via two separate USB 3.1 interfaces that were linked to two different USB controllers to allow for the maximum frame rate. The Kinect cameras were activated about 30 min prior to measurement to allow for pre-heating to achieve a more stable accuracy [14]. The Azure Kinect was set to NFOV mode to match the field of view of Kinect v2. Concurrently, a scan was executed by the FARO laser scanner to capture the reference ground truth data. About 60 s of the raw Kinect data was recorded with each scan. This process was repeated 15 times at each of the key locations.

For post-processing, the laser scan data were processed in SCENE software (FARO Technologies, Inc., Lake Mary, FL, USA) and exported in .pts format. Open-source 3D point cloud processing and editing software CloudCompare (CloudCompare software: https://www.danielgm.net/cc/, accessed on 20 March 2022) was used for the analysis of spatial data.

### 3.3. Setup and Data Collection: Flat Wall

**Figure 4:**
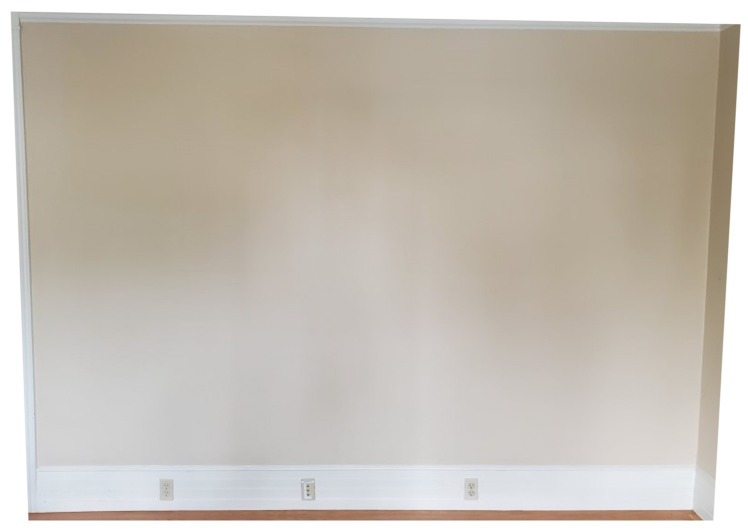
The wall used for investigating Kinect v2 and Azure Kinect sensor accuracy in horizontal and vertical directions.

A room containing a 3.45 × 2.24 m (11.3 × 7.3 ft) blank, flat wall was used to assess vertical and horizontal variations in accuracy between Kinect devices (Figure 4). The wall was separated from the floor by a trim 23 cm in height. Trim also lined the left and upper portions of the wall as well, and the right boundary was determined by its corner with a perpendicular wall.

**Figure 5:**
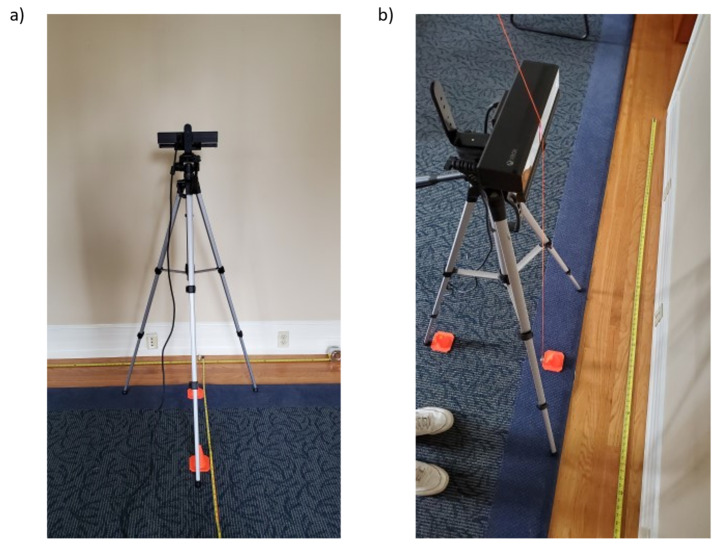
The Kinect v2 (**a**) in the 0.5 m position and (**b**) its positioning using a plumb bob.

The Azure Kinect was mounted on a tripod with its sensor at a height of 1.34 m (53 inches) such that it was aligned with the center of the wall. The sensor was then placed 0.5 m from the wall, and approximately 60 s of recording at 30 FPS was captured using Brekel PointCloud v3. This process was repeated at distances of 1, 1.5, 2, 2.5, 3, and 3.5 m. For all seven positions, the camera was aligned to point directly at the wall. Once data capture for the Azure Kinect was completed, the Kinect v2 was mounted on the same tripod and the exact same experiment was repeated (Figure 5a). A series of cones was placed on the floor at the various distances, and a plumb bob was used to align the sensors to the correct location, ensuring consistency in sensor distance across experiments (Figure 5b). Both cameras were activated about 30 min prior to measurement to allow for pre-heating.

### 3.4. Error Metrics

To properly assess the accuracy of the Azure Kinect and Kinect v2 camera systems, the data were post-processed for comparison. Even for a static scene, the Kinect devices generate a sequence of point clouds that differ dynamically from one another. Therefore, one must use separate metrics to parse out temporal and spatial errors. We call these metrics the *random depth error*, and the *systematic spatial error*, respectively.

#### 3.4.1. Random Depth Error

A random depth error metric is used to compare temporal (dynamic) errors between the two Kinect devices. This quantity is defined as

$$E_{r}=\sqrt{\frac{\sum_{i=1}^{N\;}{\left(d_{i}-\bar{d}\right)}^{2}}{N}},$$

where $d_{i}$ is the depth value of a certain pixel at time index i, N is the total number of frames, and $\bar{d}$ is the average depth value at the same pixel. The value of $E_{r}$ may be interpreted as the standard deviation of a pixel depth around its mean value. We place *N* in the denominator for the standard deviation instead of *N* − 1 in conformity with the metric used by Microsoft [18]. Since the number of samples *N* to be used is relatively large (1000), we anticipate the difference to be negligible.

#### 3.4.2. Systematic Spatial Error

A systematic spatial error metric is used to compare overall spatial errors between the two Kinect devices. Computation of this quantity requires a ground truth representation of the spatial data, which are supplied by the FARO laser scanner. In addition, we require time-averaged point clouds from the Kinect devices that are properly registered to the FARO-generated clouds to eliminate the effect of the random depth error incorporated in each depth frame. Details of how to obtain and process these point clouds follow in the next section.

A systematic spatial error metric is determined using CloudCompare’s cloud-to-cloud distance tool. The FARO cloud is selected as the “Reference” cloud, while the Kinect cloud is selected as the “Compared” cloud. This tool employs a nearest neighbor (NN) approach, wherein for each point of the reference cloud, the closest point is found in the compared cloud. To calculate the average distance between point clouds representing the same board, a point belonging to the FARO board is located by the vector $x_{j}$ (j=1,…,J), and a point belonging to the Kinect board is located by the vector $y_{k}$ (k=1,…,K). Here, J and K are the total number of points belonging to the FARO cloud and Kinect cloud, respectively. For each point $x_{j}\;$in the reference cloud, the nearest neighbor in the compared cloud is found at the index where their Euclidean distance is minimized:

$$D_{j}=\underset{k\in \left(1,\ldots ,K\right)}{\min}||x_{j}-y_{k}||.$$

This computation is repeated over all j∈(1,…,J), after which the metric for systematic spatial error is recovered as the mean of all nearest neighbor distances $D_{j}$:

$$E_{s}=\frac{\sum_{j=1}^{J}D_{j}}{J}.$$

CloudCompare reports this value in addition to its standard deviation from its cloud-to-cloud distance tool. For the computation, the Octree level is selected as “auto” and the multi-threaded 16/16 option is selected.

### 3.5. Data Processing

The raw data from the FARO laser scanner are stored in the form of .fls files, which are processed and registered in SCENE to a common coordinate system. We use Brekel PointCloud v3 software to export a sequence of depth maps from recordings of each Kinect device. These depth maps serve as the raw data, which are processed into a 3D point cloud, as detailed below.

#### 3.5.1. Random Depth Error from Depth Maps

The depth map for the Kinect v2 as exported by the Brekel software is a 512 × 424 px image of 16 bit unsigned integers that represent depth values in millimeters for each pixel. The Azure Kinect is a 640 × 576 px image of the same type of data. Since the random depth error is defined for a singular point across multiple subsequent frames, we chose to examine a point near the center of each board for comparison. We calculated $E_{r}$ across N= 1000 frames, which corresponds to a time duration of approximately 33 s.

**Figure 6:**
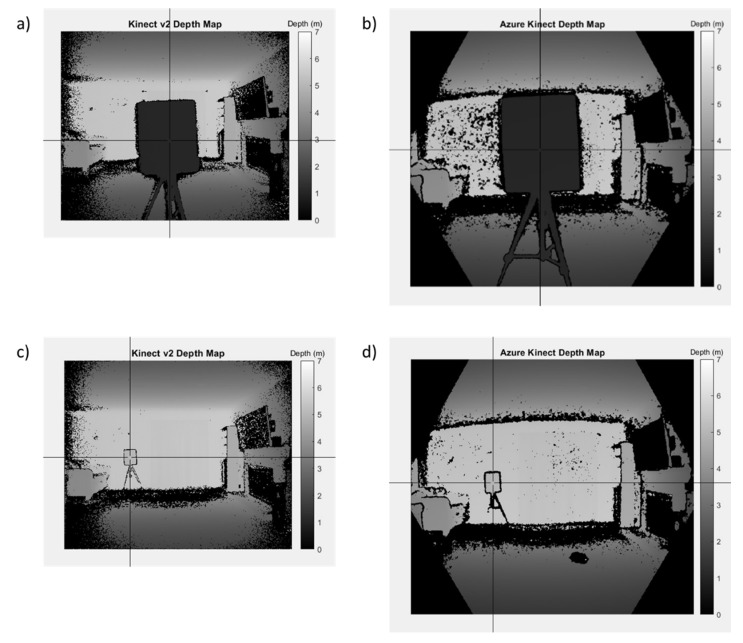
Pixel selection for computing the random depth error of the (**a**) Kinect v2 for board location 1, (**b**) Azure Kinect for board location 1, (**c**) Kinect v2 for board location 15, and (**d**) Azure Kinect for board location 15. Note that the depth maps are mirrored.

A pixel is manually selected for comparison by graphical input using MATLAB near the center of the board (Figure 6). If a zero value is returned, then there is a lack of depth data for the given pixel. Invalid pixels are ignored in the computation. We found that the center of the board is relatively immune to missing (zero) depth values compared to other areas of the image, such as the corners of the depth maps from the Kinect v2, which lack a mask as compared to the Azure Kinect. The mean and standard deviation of the depth distribution across 1000 consecutive frames for the selected pixel is then calculated to obtain the random depth error at each target location. Note that the positions of the Kinect cameras and the laser scanner were fixed during the acquisition process. At each of the five Z-depth values, we therefore obtained one sample that was close to the optical center and two samples that were offset horizontally to the left and right.

#### 3.5.2. Averaged Depth Maps and the Pinhole Camera Model

To compute the systematic spatial error, an averaged point cloud from the Kinect recordings must be generated for comparison to ground truth. By doing this, we eliminate the variability of the depth data due to time fluctuations. A MATLAB script was created to compute the average depth values of each pixel across 1000 sequential depth maps. These averaged depth maps only take into account non-zero depth values. From the averaged depth maps, a 3D point cloud is computed.

We compute the 3D point cloud corresponding to a 2D depth map using the pinhole camera model with distortion. The depth value of each pixel along with well-calibrated camera intrinsic parameters is the input to the model, while the 3D position in space of each pixel is the output. As we describe later, the practical implementation of computing the average point cloud for the two Kinects somewhat differed from this standard approach. Here, we provide details for completeness such that we may demonstrate how different parameters of the model affect the final accuracy of the 3D point cloud reconstruction.

For an image free of distortions taken with a pinhole camera, the mapping from a 2D image pixel to a 3D point is

$$X=\frac{x-c_{x}}{f_{x}}z,$$

$$Y=\frac{y-c_{y}}{f_{y}}z,$$

Z=z.

In these formulas, (x,y) are pixel coordinates relative to the top left corner of the image, where x is positive rightward and y is positive downward, $\left(c_{x},c_{y}\right)$ is the location of the camera center (i.e., center of projection), $f_{x}$ and $f_{y}$ are camera focal lengths, and z is the measured depth value from the Kinect device. The Kinect provides the depth map as a 16 bit image where the intensity value of each pixel I(x,y) corresponds to the acquired distance z in millimeters. The origin for the X, Y, and Z coordinates is at the focal point of the infrared sensor. X and Y are oriented in the same manner as x and y pixel coordinates following the computer vision convention, while the direction of Z is given by the right-hand rule. If the real focal length of the camera lens is f, the sensor width is W, the sensor height is H, the number of pixels in horizontal dimension is M, and the number of pixels in vertical dimension is N, and then a scaled focal length can be defined in each of the image dimensions as

$$f_{x}=f\frac{M}{W},\;f_{y}=f\frac{N}{H}.$$

Therefore, it is implied that $x,y,c_{x},$ and $c_{y}$ are in units of pixels.

The depth maps are also subject to optical distortions of the lens system in front of the infrared camera. These effects can be modeled with different lens distortion models. The recent implementation in Azure Kinect SDK uses the standard Brown–Conrady model with radial and tangential distortion terms [19]. The formulation of the parameters matches the implementation of the distortion model used in the computer vision library OpenCV (OpenCV Library, https://opencv.org, accessed on 20 March 2022). If $x_{d}$ and $y_{d}$ are image coordinates in the *distorted* image space, then the following relations apply between distorted and undistorted pixel coordinates:

$$x_{d}=\frac{1+k_{1}r^{2}+k_{2}r^{4}+k_{3}r^{6}}{1+k_{4}r^{2}+k_{5}r^{4}+k_{6}r^{6}}x+2p_{1}xy+p_{2}\left(r^{2}+2x^{2}\right),$$

and

$$y_{d}=\frac{1+k_{1}r^{2}+k_{2}r^{4}+k_{3}r^{6}}{1+k_{4}r^{2}+k_{5}r^{4}+k_{6}r^{6}}y+2p_{2}xy+p_{1}\left(r^{2}+2y^{2}\right).$$

In these non-linear mappings, $k_{1},k_{2},k_{3},k_{4},k_{5},$ and $k_{6}$ are the radial distortion coefficients, $p_{1}$ and $p_{2}$ are the tangential distortion coefficients (also referred to as decentering parameters), and r is the radial distance from the distortion center, assumed to be at the center of projection $\left(c_{x},c_{y}\right)$:

$$r=\sqrt{{\left(x-c_{x}\right)}^{2}+{\left(y-c_{y}\right)}^{2}}.$$

When generating the point cloud, a reverse mapping has to be performed to obtain undistorted pixel coordinates from the observed (distorted) pixel values. Since there is no analytical solution to the above equations, the distortion equations must be solved numerically for each value of x and y given the distorted pixel location $\left(x_{d},y_{d}\right)$.

#### 3.5.3. Propagation of Errors

While the random error describes the variation in a direct measurement that is obtained using ToF principles, the systematic error is strongly dependent on the accuracy of many calibration parameters. If x and y are coordinates in the undistorted image, then a propagation of errors yields

$$\delta X=-\frac{z}{f_{x}}\delta c_{x}-\frac{\left(x-c_{x}\right)z}{f_{x}^{2}}\delta f_{x}+\frac{x-c_{x}}{f_{x}}\delta z,$$

$$\delta Y=-\frac{z}{f_{y}}\delta c_{y}-\frac{\left(y-c_{y}\right)z}{f_{y}^{2}}\delta f_{y}+\frac{y-c_{y}}{f_{y}}\delta z,$$

δZ=δz.

The quantities $\delta f_{x},\delta f_{y},\delta c_{x},$ and $\delta c_{y}$ may be considered as uncertainties or errors in the calibration parameters, while δz is considered to be the depth error of the measurement. Hence, the accuracy of reconstructed point cloud’s X and Y locations is highly dependent on the calibration accuracy of the camera intrinsics $f_{x},f_{y},c_{x},$ and $c_{y}$ in addition to any measurement error in z (i.e., depth). In contrast, the accuracy of the point cloud Z location depends only on the measurement error in z.

In addition, the non-linear mapping from radial and tangential distortions also contributes to the error in X and Y through any errors in the distortion coefficients [20]. The computation is too unwieldy to describe here. However, one can expect the contribution to the overall error to increase significantly toward the boundaries of the image where distortion may be higher (see Figure 6). We conclude that the systematic spatial error is highly dependent on the quality of the intrinsics obtained from camera calibration. In our assessment, we do not specifically calibrate the Kinect system but rather use the calibration values encoded into each camera unit that are accessible via their respective Software Development Kit (SDK).

#### 3.5.4. Computation of the Time-Averaged Point Cloud

For the Azure Kinect camera, the Azure Kinect SDK provides the camera model parameters that correspond to the aforementioned pinhole camera model with the radial and tangential lens distortions. To compute the 3D point cloud from an average depth image, we queried the intrinsic camera parameters from the SDK and computed the point cloud using custom OpenCV code. The point cloud was then saved in a standard .ply format for further processing.

Although the Kinect v2′s Kinect for Windows SDK 2.0 provides access to the depth camera calibration parameters through the *CameraIntrinsics* data structure (including two focal lengths, camera center, and the first three radial distortion parameters), the documentation recommends the use of the *CoordinateMapper* interface to accurately recover a 3D point from pixel coordinates. To use the *CoordinateMapper* for offline post-processing of the average depth images, we wrote a custom C++ application that loaded the raw depth image from a file, connected to the Kinect camera, and then passed the depth buffer of the input image to the *CoordinateMapper* method *MapDepthFrameToCameraSpace*. Since each manufactured camera has a unique calibration, we used the same Kinect unit that was deployed for the initial data collection.

For both Kinect systems, we confirmed that the Brekel PointCloud software uses the same approach to produce the point cloud data by comparing their 3D point cloud with our recovered point cloud for a single-capture depth data frame.

#### 3.5.5. Point Cloud Processing

**Figure 7:**
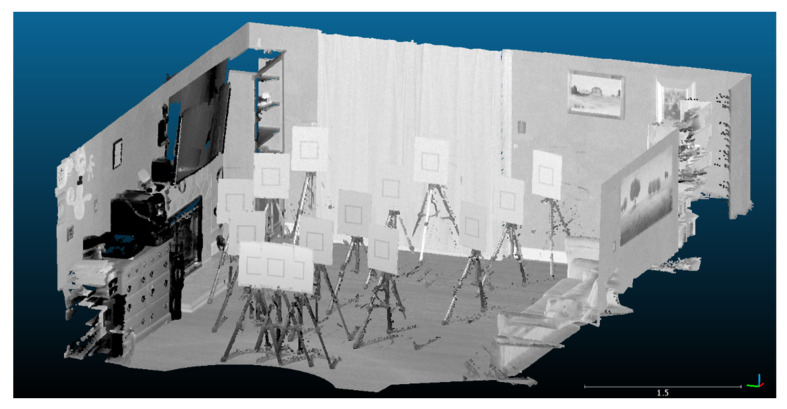
All fifteen FARO scans are registered relative to one another in the static scene.

**Figure 8:**
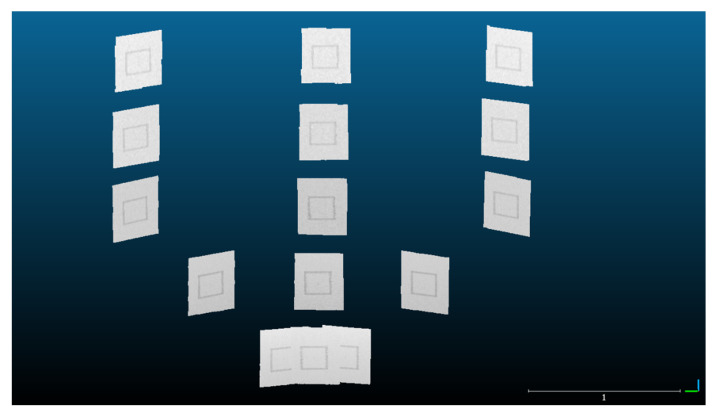
Each FARO scan is clipped to only contain the sample board. Edge artifacts are trimmed from the boundaries of each board.

The raw scans from the FARO laser scans were registered using SCENE software (Figure 7). The maximum point error, mean point error, and minimum overlap were reported by the software as 0.4 mm, 0.4 mm, and 93.8%, respectively. Each FARO scan was then segmented so as to only include the board itself (Figure 8). Edge artifacts were segmented out to minimize error in the comparison to the Kinect-generated clouds.

**Figure 9:**
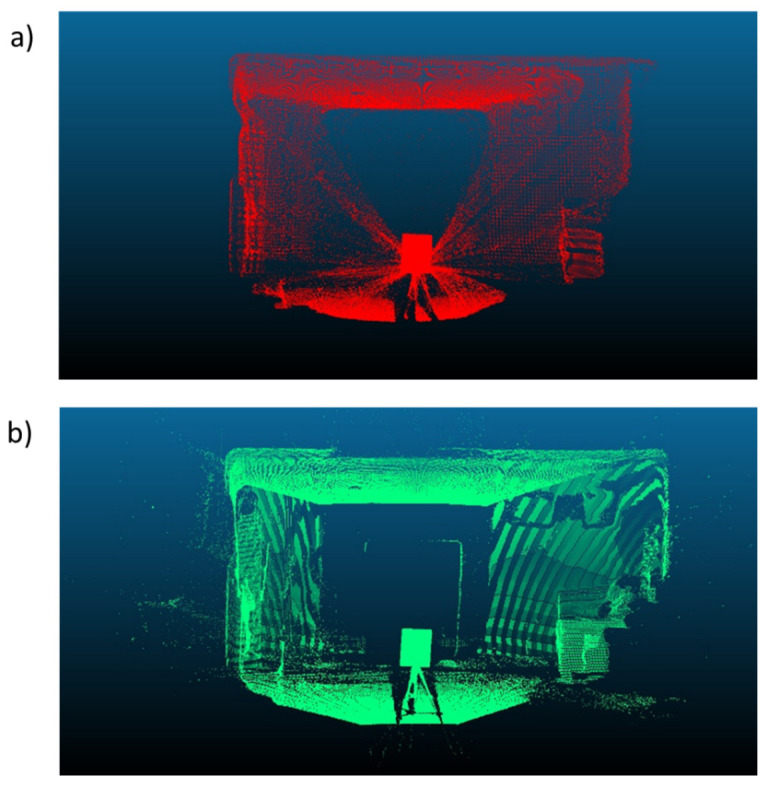
The point cloud generated from averaging 1000 consecutive depth frames for the (**a**) Kinect v2 and (**b**) Azure Kinect for the first board location. The smearing of points outward from the origin for the Kinect v2 is due to a lack of mask for the Kinect v2.

Next, an averaged point cloud was generated for 1000 frames of the Azure Kinect and the Kinect v2, as detailed in Section 3.5.2 and Section 3.5.4 (Figure 9). We note that there was a smearing of 3D points outward from the origin when processing the Kinect v2 data due to the poor quality of the depth map toward the corners of the image. The Azure Kinect avoids issues at the image corners by applying a mask on the depth map (Figure 6). As in the case of the FARO clouds, the two sets of Kinect clouds were segmented to only include each board without edge artifacts. The point clouds generated from each Kinect individually share a common coordinate system, and no registration was needed within each set.

**Figure 10:**
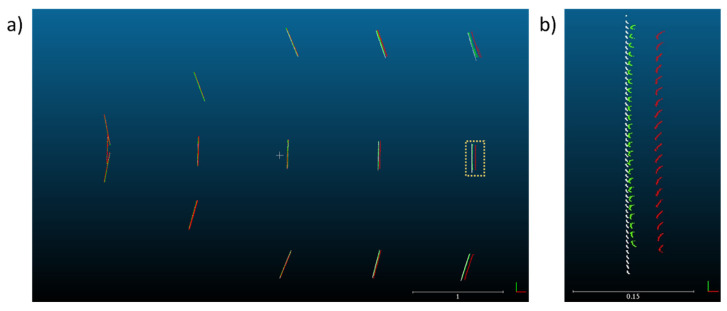
(**a**) A top view of the FARO (black and white), Kinect v2 (red), and Azure Kinect (green) point cloud sets after registration and segmentation. (**b**) A magnified view of board location 5 displaying the three point sets. The scale bar is in units of meters.

To register each Kinect-generated cloud to the FARO cloud, a distance-preserving transformation was applied that roughly placed the scans in place. Then, the Fine Registration (ICP) procedure was used in CloudCompare on board location 1 (the closest central point of the grid arrangement as shown in Figure 3), which was assumed to be the most accurate location. The corresponding transformation was copied and applied to each individual board within each Kinect dataset, so as to rigidly transform the Kinect point clouds as a whole onto the FARO cloud coordinate space (Figure 10).

## 4. Results

**Figure 11:**
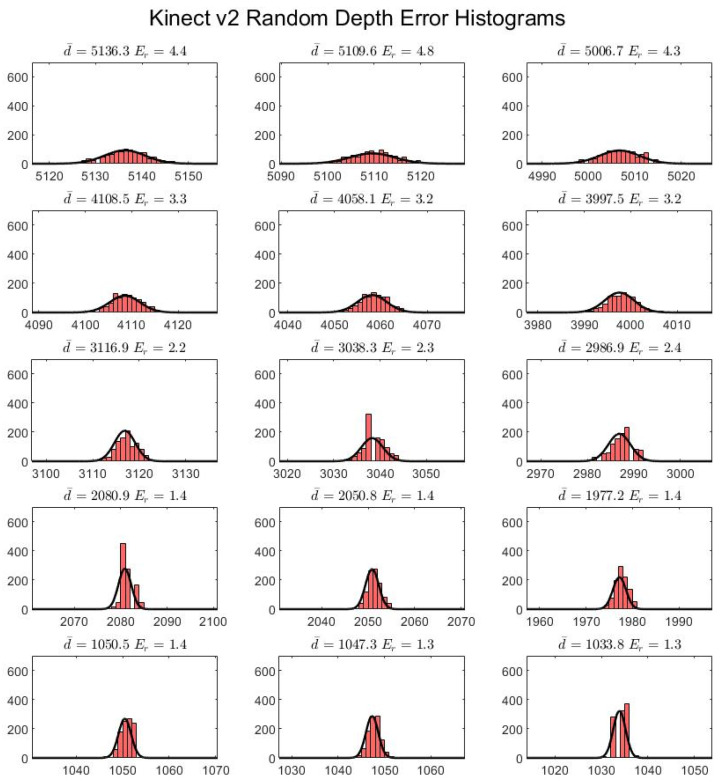
Random depth error for the Kinect v2, where $\bar{d}$ indicates the average depth value and $E_{r}$ is the standard deviation, or the random depth error. The horizontal axes and titles are in units of millimeters, while the vertical axes indicate bin counts. Each bin is 1 mm in width.

**Figure 12:**
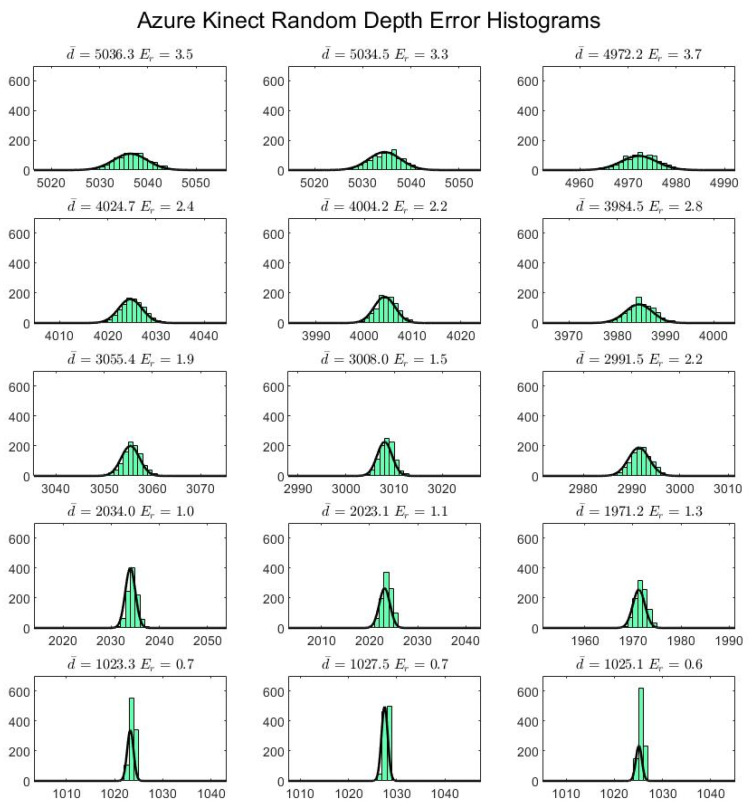
Random depth error for the Azure Kinect, where $\bar{d}$ indicates the average depth value and $E_{r}$ is the standard deviation, or the random depth error. The horizontal axes and titles are in units of millimeters, while the vertical axes indicate bin counts. Each bin is 1 mm in width.

The random depth error is plotted as a series of histograms for each board location and each sensor (Figure 11 and Figure 12). A 1 mm bin width size is used to classify the data. The results appear to show a Gaussian distribution in the case of both sensors for every board location.

**Figure 13:**
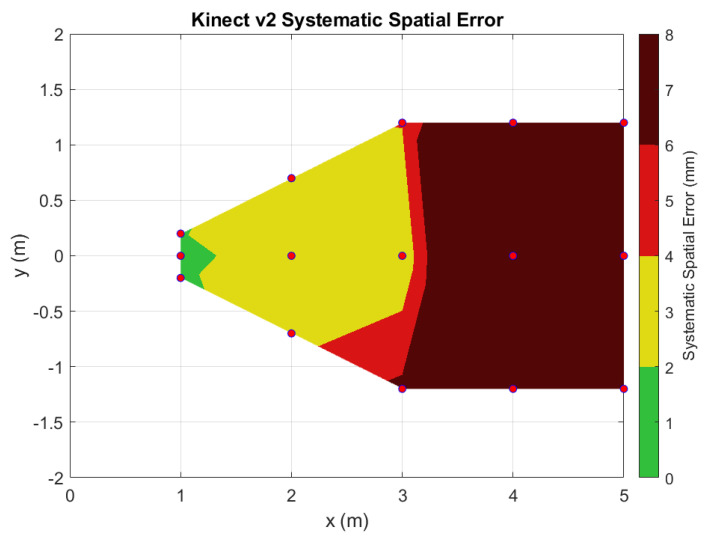
Contour plot of the systematic spatial error over the horizontal plane for the Kinect v2 sensor.

**Figure 14:**
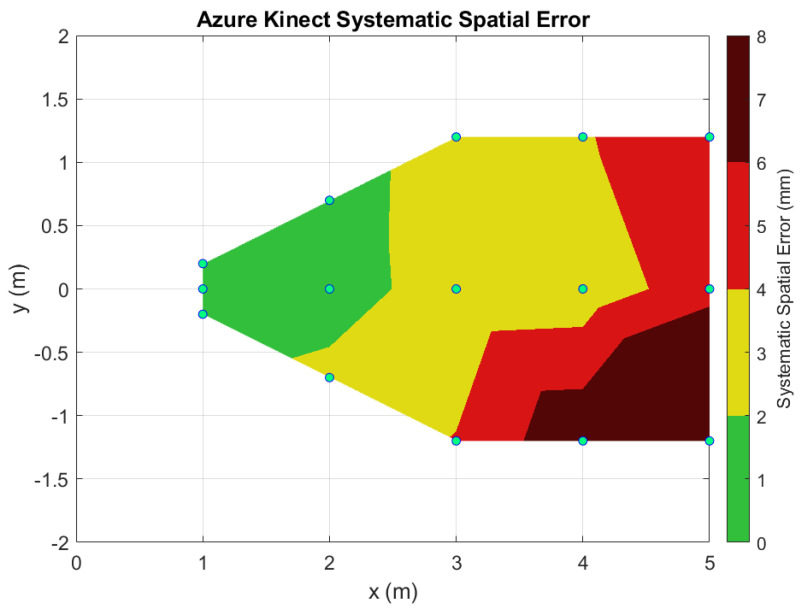
Contour plot of the systematic spatial error over the horizontal plane for the Azure Kinect sensor.

The systematic spatial errors and their standard deviations are logged into Table 2. Using this data, a contour plot over the horizontal plane was generated from the key locations to visualize the error distribution across the range for both the Kinect v2 (Figure 13) and the Azure Kinect (Figure 14). To generate these plots, a mesh grid was created using Delaunay triangulation. The data were then linearly interpolated using MATLAB’s *griddata* function and displayed as a surface plot.

**Figure 15:**
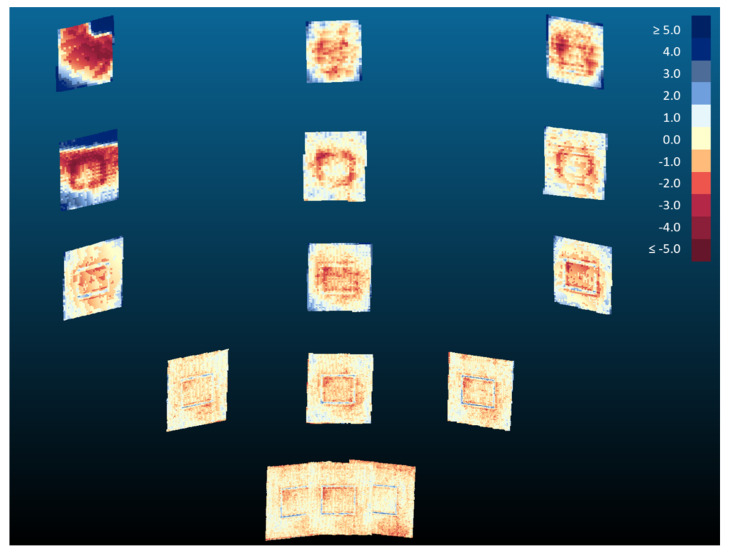
Color map distance plots of the averaged Kinect v2 point clouds to their planes of best fit. Distance values are in millimeters.

**Figure 16:**
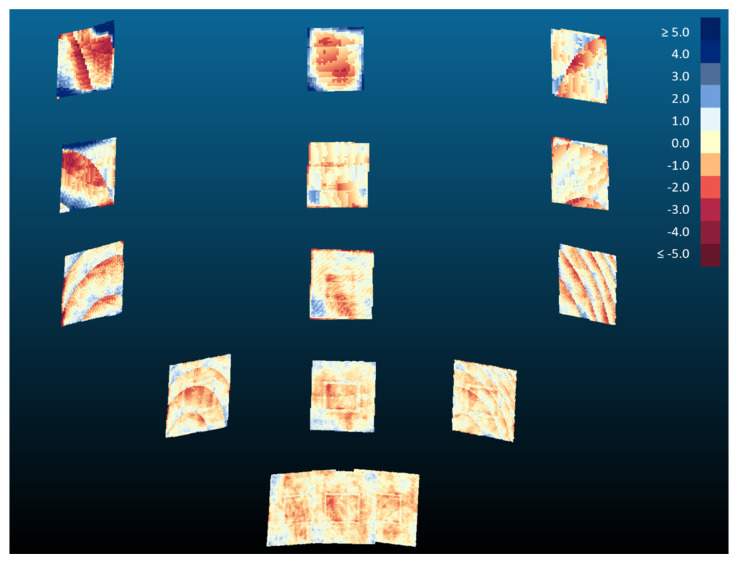
Color map distance plots of the averaged Azure Kinect point clouds to their planes of best fit. Distance values are in millimeters.

By fitting a plane to the Kinect v2 and Azure Kinect averaged point clouds, we were also able to investigate the planarity of the data. Using a signed distance metric, a color map of distances to the plane of best fit was generated for each board location (Figure 15 and Figure 16). The color scale is set to display the range of distance errors with colors saturating to dark red at −5 mm and to dark blue at 5 mm. Yellow colors are associated with points laying on the plane of best fit. A negative value (reddish colors) indicates a point in front of the plane, while a positive value (bluish colors) indicates a point behind the plane.

**Figure 17:**
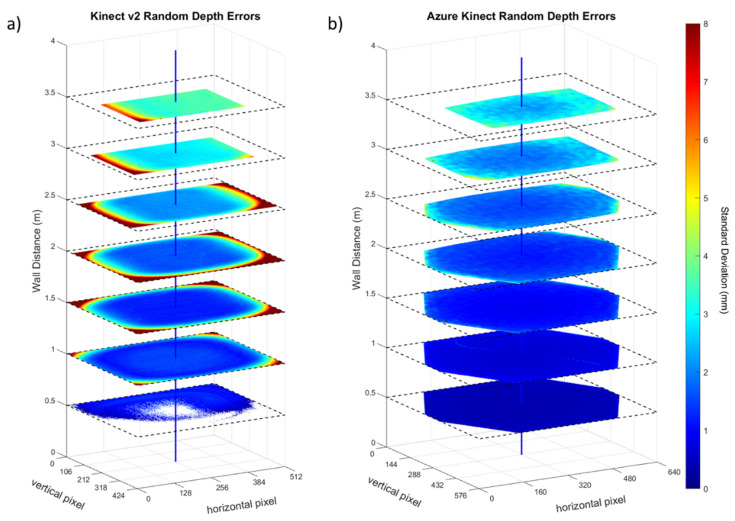
Distribution of the random depth error (mm) at varying distances to a flat wall for the (**a**) Kinect v2 and (**b**) Azure Kinect. The observed flat area was cropped at larger distances to retain the same portion of the scene in all views.

The random depth error distribution across horizontal and vertical directions for each Kinect sensor at various distances from 0.5 to 3.5 m is displayed in Figure 17. For each pixel of the image plane, the error is computed as a standard deviation across 1000 frames, which is the random depth error metric of Section 3.4.1. The dark blue colors represent low errors (0 to 2 mm), while the orange and red colors represent high errors (5 mm or more). The line penetrating the image centers is displayed in blue. At larger distances (beyond 2 m), the area of the wall was cropped in the depth image to retain the same portion of the scene in all views since the wall was not large enough to fully cover the entire field of view.

## 5. Discussion

As our results demonstrate, the Azure Kinect has improved spatial and temporal accuracy over the Kinect v2 (Figure 10, Figure 11, Figure 12, Figure 13 and Figure 14). In particular, the random depth error can be seen to have been halved from the Kinect v2 to the Azure Kinect for distances in the range of 0.0 to 1.0 m from the camera. For distances farther away, the Azure Kinect still reigns supreme compared to its predecessor for all board locations. In terms of the systematic spatial error, our analysis indicates that the Azure Kinect has extended the range for higher accuracy measurements with systematic error under 2 mm from the 1.0 to the 2.0 m range. Depending on the application, measurements in the range of up to 4.0 m may be acceptable as well.

Since we followed the target placement based on the Yang et al. [7] paper, we can compare our results for Kinect v2′s systematic error with Yang’s. Their systematic error was reported to be 0 to 2 mm in the central region of the viewing cone for the distances from 0.5 to 3.0 m. Our systematic error for Kinect v2 was somewhat larger, 0 to 4 mm in the same range. A possible reason for these differences may be due to the nature of the ground truth measurement. Yang used a digital laser pointer that has an accuracy of ±2 mm for a single point distance measurement, as reported in their paper. Conversely, our Kinect measurements were compared against a laser scanned geometry of a planar patch, where the laser scanner had a calibrated uncertainty of about ±0.5 mm.

At distances of 3 m and more, the Kinect v2 overestimated depth values compared to the FARO laser scans (Figure 10). This measurement error is further exacerbated for positions away from the optical center of the Kinect v2, as X and Y point data are derived from Z-depth values using the formulas from Section 3.5.2. The source of this depth error may be due to imperfections in the compensation of various ToF limitations, including systematic errors, object boundary ambiguity, multipath error, and phase wrapping [21].

When comparing our results with the hardware specifications of the Azure Kinect [10], we observed random errors to be in the range of 0.6 to 3.7 mm (standard deviation) for distances from 1.0 to 5.0 m, which is considerably less than the cited 17 mm. However, our error analysis only incorporated the center of the target surface, whereas the largest random errors are typically observed toward object boundaries. In addition, our systematic spatial errors ranged from 1.1 to 12.7 mm for the range of 1.0 to 5.0 m, which is less than the maximum expected 12 to 16 mm systematic depth error at this range. We note that our systematic spatial error metric should serve as an upper limit to the systematic depth error reported by Microsoft. For the Kinect v2, no official performance accuracy values are available.

The two sensors are more capable of maintaining board planarity at closer distances (Figure 15 and Figure 16). Measurements of a planar object at farther distances are more susceptible to warping at board corners. In our investigation of planarity, we also uncovered an interesting phenomenon whereby the patterns of the infrared radiation supplied by the emitters were evident in board locations 8 and 13 for the averaged point clouds of the Azure Kinect. These board locations are the farthest from the optical axis in the infrared image plane. We noted that this pattern was not present for the Kinect v2. We can only speculate that this phenomenon is due to differences in emitter characteristics and placement between the Azure Kinect and the Kinect v2 (see the arrangement of red lights in Figure 1b).

The experiment involving the flat wall examined the distribution of depth error across the field of view when observing a flat surface at various distances (Figure 17). At the closest distance of 0.5 m, the Kinect v2 only partially captured the wall surface, likely due to the saturation of the infrared sensor at this close range. Farther away from the wall, the Kinect v2 exhibits relatively uniform error close to the optical axis with the error increasing radially closer to the edges of the image. The depth errors in the region around the center of the image remain under about 4 mm, which is consistent with the observations in the first experiment (Figure 11).

As mentioned earlier, the depth map acquired in the Azure Kinect inherently has an octagonal mask, which blocks the pixels toward the edges of the image plane. The Azure Kinect is also able to capture a coherent depth map at the closest range of 0.5 m, indicating that the valid range has been extended for nearer distances. From 2.5 to 3.5 m, the Azure Kinect shows a marked improvement in accuracy for measurements close to the optical axis as compared to the Kinect v2, indicating the valid range has been extended for farther distances for objects captured close to the optical axis. The depth error in the region around the optical axis remains under 2.5 mm even at the maximal observed distance of 3.5 m. This finding is consistent with the observations in the first experiment (Figure 12).

Azure Kinect and Kinect v2 depth measurements away from the optical center are influenced by several factors, including optical distortions of the lens, IR illuminator irregularities [4,21], and the accuracy of the overall calibration. Hence, one should proceed with caution in interpreting data if objects are placed off-center in the frame of view, especially for Kinect v2.

For the Azure Kinect, the ideal location for measurement when using the entire field of view is within 2.5 m of the sensor. Beyond 2.5 m, the object to be captured should be close to the optical axis. This is particularly relevant when capturing large objects such as the human body from head to toe, whether for body scanning or dynamic motion tracking (via the Azure Kinect Body Tracking SDK). For these applications, either the NFOV or WFOV setting should be chosen accordingly.

The presented study has several limitations. Our accuracy assessment was performed using only a single unit of each Kinect. Future studies may consider additional variables and parameters to improve testing. For example, the board and easel were placed roughly above each cone. A more precise measurement could entail marking a point on the board for observation. In addition, more defined objects could be placed throughout the testing space to allow for a more accurate alignment of the point clouds and automatic segmentation during post-processing. A common method for achieving this with multiple laser scans is by using a reference sphere set.

In summary, our accuracy assessment for the Kinect v2 camera overall compares with other studies. The results for the Azure Kinect indicate that the camera outperforms the Kinect v2 in accuracy and operating distance for similar capture volume. Our results also confirmed that the systematic and random errors in NFOV mode fall within the given specifications. The results suggest that the Azure Kinect is a suitable substitute for 3D scanning applications that were previously based on the Kinect v2.

## Figures and Tables

**Table 1:** Comparison of the performance characteristics of the three systems: Microsoft Kinect v2, Microsoft Azure Kinect DK, and the FARO X330 HDR laser scanner. An asterisk (*) indicates binned depth mode.

Parameter	Microsoft Kinect v2	Microsoft	FARO X330 HDR Laser Scanner
		Azure Kinect DK	
Operation Principle	ToF with modulation up to 130 MHz	ToF with modulation 200–300 MHz	Infrared laser beam (1550 nm wavelength) with rotating mirror
Color Resolution	1920 × 1080 px (2 Mpx)	4096 × 3072 px (12.6 Mpx)	Up to 170 Mpx color, High Dynamic Range (HDR)
Color FOV (H × V)	84.1° × 53.8°	90° × 59°	Up to
			360° × 300°
Depth Resolution	512 × 424 px (0.22 Mpx)	WFOV:	Up to 710.7 MPts
		1024 × 1024 px (1MPx)	
		512 × 512 px * (0.26 Mpx)	
		NFOV:	
		640 × 576 px (0.37 Mpx)	
		320 × 288 px * (0.09 Mpx)	
Depth FOV (H × V)	70.6° × 60°	WFOV:	360° × 300°
		120° × 120°	
		NFOV:	
		75° × 65°	
Depth Range	0.5–4.5 m	WFOV:	0.6–300 m
		0.25 to 2.21 m	
		0.25 to 2.88 m *	
		NFOV:	
		0.50 to 3.86 m	
		0.50 to 5.46 m *	
Acquisition Rate	30 Hz	30 Hz	5–15 min

**Table 2:** Mean and standard deviation values of the systematic spatial error at each location for both Kinect v2 and Azure Kinect. Lowest values for a particular location are marked in bold.

ID	Whiteboard *X* (m)	Whiteboard *Y* (m)	Kinect v2	Kinect v2	Azure Kinect	Azure Kinect
			Systematic Spatial Error (mm)	Standard Deviation (mm)	Systematic Spatial Error (mm)	Standard Deviation (mm)
1	1	0.0	1.2	1.1	**1.1**	**0.9**
2	2	0.0	3.7	1.2	**1.4**	**0.6**
3	3	0.0	**2.3**	1.6	2.6	**1.2**
4	4	0.0	18.7	1.1	**2.8**	**0.9**
5	5	0.0	39.4	1.7	**5.1**	**1.4**
6	1	0.2	1.9	1.8	**1.3**	**1.3**
7	2	0.7	3.1	1.7	**1.5**	**0.6**
8	3	1.2	4.1	2.5	**2.6**	**1.0**
9	4	1.2	14.3	2.2	**3.8**	**1.4**
10	5	1.2	37.1	3.0	**5.7**	**1.4**
11	1	−0.2	1.7	2.2	**1.3**	**1.3**
12	2	−0.7	3.2	**1.9**	**2.3**	2.9
13	3	−1.2	6.4	**1.6**	**4.1**	2.7
14	4	−1.2	19.8	3.3	**7.7**	**2.3**
15	5	−1.2	47.7	5.6	**12.7**	**3.3**

## Data Availability

All data generated or appeared in this study are available upon request by contact with the corresponding author.
